# Abaloparatide in Postmenopausal Women With Osteoporosis and Type 2 Diabetes: A Post Hoc Analysis of the ACTIVE Study

**DOI:** 10.1002/jbm4.10346

**Published:** 2020-02-27

**Authors:** Ruban Dhaliwal, Didier Hans, Gary Hattersley, Bruce Mitlak, Lorraine A Fitzpatrick, Yamei Wang, Ann V Schwartz, Paul D Miller, Robert G Josse

**Affiliations:** ^1^ Metabolic Bone Disease Center State University of New York Upstate Medical University Syracuse NY USA; ^2^ Center of Bone Disease, Bones & Joints Department Lausanne University Hospital Lausanne Switzerland; ^3^ Clinical Development, Radius Health, Inc. Waltham MA USA; ^4^ Biostatistics, Radius Health, Inc. Waltham MA USA; ^5^ Department of Epidemiology and Biostatistics UCSF School of Medicine San Francisco CA USA; ^6^ Research, Colorado Center for Bone Research Lakewood CO USA; ^7^ Research, St. Michael's Hospital University of Toronto Toronto Canada

**Keywords:** ABALOPARATIDE, ANABOLICS, BONE MINERAL DENSITY, CLINICAL TRIALS, DXA, FRACTURE PREVENTION, OSTEOPOROSIS, TRABECULAR BONE SCORE, TYPE 2 DIABETES MELLITUS

## Abstract

Type 2 diabetes mellitus (T2DM) increases fracture risk despite normal or increased BMD. Abaloparatide reduces fracture risk in patients with postmenopausal osteoporosis (PMO); however, its efficacy in women with T2DM is unknown. This post hoc analysis evaluated the efficacy and safety of abaloparatide in patients with T2DM. The analysis included patients with T2DM from the **A**baloparatide **C**omparator **T**rial **I**n **V**ertebral **E**ndpoints (ACTIVE), a phase 3, double‐blind, randomized, placebo‐ and active‐controlled trial. In ACTIVE, participants were randomized 1:1:1 to daily s.c. injections of placebo, abaloparatide (80 μg), or open‐label teriparatide (20 μg) for 18 months. A total of 198 women with PMO and T2DM from 21 centers in 10 countries were identified from ACTIVE through review of their medical records. The main outcomes measured included effect of abaloparatide versus placebo on BMD and trabecular bone score (TBS), with secondary outcomes of fracture risk and safety, in patients from ACTIVE with T2DM. Significant (*p* < 0.001) improvements in BMD at total hip (mean change 3.0% versus −0.4%), femoral neck (2.6% versus −0.2%), and lumbar spine (8.9% versus 1.3%) and TBS at lumbar spine (3.72% versus −0.56%) were observed with abaloparatide versus placebo at 18 months. Fracture events were fewer with abaloparatide treatment in patients with T2DM, and differences were not significant between groups except nonvertebral fractures in the abaloparatide versus placebo groups (*p* = 0.04). Safety was consistent with the ACTIVE population. In conclusion, in women with PMO and T2DM, abaloparatide treatment resulted in significant improvements in BMD and TBS versus placebo, consistent with the overall ACTIVE population © 2020 The Authors. *JBMR Plus* published by Wiley Periodicals, Inc. on behalf of American Society for Bone and Mineral Research.

## Introduction

Osteoporosis significantly increases the risk of fragility fractures, which can substantially impact affected individuals and their families because of increased mortality, morbidity, and loss of independence and, thereby, imposes a high economic burden on society.^1^, ^2^, ^3^ Osteoporosis and type 2 diabetes mellitus (T2DM) are frequently comorbid conditions, both of which increase in prevalence with age.^4^, ^5^ Compared with the general population, patients with T2DM have a greater risk of major osteoporotic fractures including hip fractures.^6^, ^7^, ^8^ T2DM is also a risk factor for delayed healing and increased mortality following a hip fracture.^9^, ^10^ Because of these issues, effective fracture prevention strategies are needed for patients with T2DM.^11^

The increased risk of fracture in patients with T2DM is somewhat paradoxical, as these individuals often exhibit normal or increased BMD.^12^, ^13^, ^14^ Evidence suggests that fracture risk in patients with T2DM is associated with bone fragility caused by deterioration in bone quality.^15^, ^16^, ^17^ Trabecular bone score (TBS) is strongly correlated with microarchitectural parameters that reflect bone strength and is lower in patients with T2DM, particularly those with poor glycemic control (HbA1c ˃7.5%) compared with the general population.^18^, ^19^, ^20^ Recent guidelines support the use of TBS to help predict fracture risk in combination with other fracture risk assessment strategies, including in patients with T2DM.^21^

Several potential consequences of hyperglycemia have been postulated to contribute to altered bone metabolism, including accumulation of advanced glycation end products in collagen, increased IL‐6 production leading to stimulation of osteoclasts, osmotic‐induced damage to osteoblasts, suppression of gene expression involved with osteoblast maturation, and potential effects of microvascular disease on bone.^17^, ^22^, ^23^, ^24^ In particular, T2DM is characterized by decreased bone turnover, reduced PTH levels, lower circulating levels of bone formation markers, increased circulating sclerostin, and increased cortical pore volume.^15^, ^16^, ^25^, ^26^

In summary, bone in T2DM appears to be characterized by relatively preserved BMD with deficits in other aspects of bone quality. This combination has led to concerns that antiresorptive osteoporosis medications, which target prevention of bone loss to reduce fracture risk, may not be as effective in T2DM patients. On the other hand, the low bone turnover associated with T2DM suggests that anabolic agents, which stimulate bone formation and improve microarchitecture, may be beneficial in patients with T2DM and should be further investigated in this population.

Abaloparatide is a selective activator of the parathyroid 1 receptor (PTH1R) signaling pathway that favors the stimulation of bone formation.^27^ In preclinical and clinical studies, abaloparatide increased BMD.^28^, ^29^, ^30^, ^31^, ^32^ improved bone microarchitecture,^31^, ^32^, ^33^ and increased bone strength.^28^, ^31^ In ACTIVE (**A**baloparatide **C**omparator **T**rial In **V**ertebral **E**ndpoints), the pivotal phase 3 study for abaloparatide in women with postmenopausal osteoporosis, abaloparatide significantly increased BMD and decreased the risk of new vertebral, nonvertebral, clinical, and major osteoporotic fractures versus placebo, and decreased risk of major osteoporotic fractures versus teriparatide.^30^

In this post hoc analysis, we evaluated the efficacy and safety of abaloparatide in the subgroup of ACTIVE patients with T2DM. Because of sample size limitations, our analysis primarily focused on efficacy with regard to BMD and TBS. Secondarily, we assessed treatment effects on fracture rates.

## Participants and Methods

### Study design

The design of the ACTIVE study (NCT01343004) has been previously described.^30^ Briefly, ACTIVE was a randomized, double‐blind, placebo‐ and active‐controlled, multicenter trial (21 centers in 10 countries) to evaluate the efficacy and safety of abaloparatide for the prevention of fracture in postmenopausal women with osteoporosis. The 18‐month study enrolled postmenopausal (≥5 years) women aged 49 to 86 years with osteoporosis. Women were eligible for the study if they had a BMD *T*‐score ≤ −2.5 and >−5.0 at the lumbar spine or femoral neck and radiological evidence of ≥2 mild or ≥1 moderate lumbar or thoracic vertebral fractures, or history of low trauma forearm, humerus, sacral, pelvic, hip, femoral, or tibial fracture within the past 5 years. Postmenopausal women aged ˃65 years who met the fracture criteria, but had a *T*‐score ≤ −2.0 and >−5.0, and women ˃ 65 years who did not meet the fracture criteria if they had a *T*‐score ≤ −3.0 and >−5.0, were also eligible. Additional eligibility criteria included a BMI of 18.5 to 33 kg/m^2^, inclusive; and albumin‐adjusted serum calcium, PTH (1 to 84), serum phosphorus, and alkaline phosphatase values all within the normal range during the screening period.

There were 2463 postmenopausal women randomized 1:1:1 to receive daily s.c. injections of abaloparatide (80 μg/d), matching placebo, or teriparatide (20 μg/d) for 18 months. Abaloparatide and matching placebo were administered in a double‐blind fashion. Teriparatide could only be delivered using a trademarked injection pen; so it was given as an open‐label medication.

The study was approved by the ethics committee at every participating institution and was conducted according to the recommendations of Good Clinical Practice and the Declaration of Helsinki. All patients provided written informed consent prior to participation in the study.

### Post hoc analysis of the population with T2DM

Patients from ACTIVE with T2DM were identified by review of self‐reported and physician assessed medical history. Patients with the following MedDRA (version 17.1) preferred terms^34^ in their medical history data at baseline were included in the analysis: “type 2 diabetes mellitus,” “diabetes mellitus,” and “insulin‐requiring type 2 diabetes mellitus.” Terms with “type 1 diabetes mellitus” were excluded. Of the 2463 patients enrolled in the ACTIVE trial, 198 had T2DM at ACTIVE baseline (based on standardized MeDRA queries [SMQ]). Elevated fasting glucose alone was not used to identify T2DM. Twenty‐nine patients with elevated fasting plasma glucose at baseline in ACTIVE were not included in the T2DM post hoc analysis as the MedDRA (version 17.1) preferred terms used to identify patients as having T2DM for this analysis were not included in their medical history records.

### Study outcomes

The primary objectives of this post hoc analysis were to evaluate the efficacy and safety of abaloparatide in patients from the ACTIVE trial with T2DM (*N* = 198).

Total hip, femoral neck, and lumbar spine BMD were assessed in the intent‐to‐treat (ITT) population at baseline and at months 6, 12, and 18 (BioClinica‐Synarc; Newark, CA, USA). Lumbar spine TBS was evaluated at baseline and at months 6 and 18. Patients who had their initial BMD measurement on a TBS‐compatible DXA scanner (*N* = 182) were eligible for inclusion in the TBS analysis. TBS was calculated using a modified TBS Calculator (v2.2), which considered soft tissue thickness in the algorithm rather than BMI (Medimaps Group, Plan‐les‐Ouates, Geneva, Switzerland). Percent change from baseline in lumbar spine, hip, and femoral neck BMD through the end of the 18‐month treatment period and percent change in lumbar spine TBS at the end of the 18‐month treatment period were calculated.

New vertebral fracture incidence was evaluated using the modified ITT population, which included all ITT patients who had both pretreatment and postbaseline spine X‐rays. Anteroposterior and lateral radiographs of the lumbar and thoracic spine were taken at baseline and after 18 months (end of treatment). All radiographs were assessed by a blinded, independent radiologist (BioClinica‐Synarc) and graded based on a standardized grading scale of severity of the vertebral deformity using the semiquantitative technique described by Genant and colleagues.^35^ Radiographs in which an incident fracture was identified were confirmed by a second radiologist. In case of disagreement, a third consensus assessment was made to adjudicate the incident vertebral fracture.

Additional endpoints included time to first incidence of nonvertebral fracture, clinical fracture, major osteoporotic fracture, and wrist fracture. Definitions of nonvertebral, clinical, and major osteoporotic fractures have been previously described.^30^ Wrist fractures could be included in nonvertebral fractures, clinical fractures, and major osteoporotic fractures and were also analyzed separately. Incidence of nonvertebral, clinical, major osteoporotic, and wrist fractures were evaluated using the ITT population.

Blood glucose levels were monitored during the study. Blood and urine samples were obtained under fasting conditions (8 hours) in the morning of each scheduled study visit and were collected prior to injection of the study medication during the treatment period. Safety assessments included incidence and severity of adverse events (AEs) from baseline through the 30‐day follow‐up period.

### Statistical analysis

Statistical analysis was done as previously described.^30^ Briefly, the analysis of covariance model was used to compare percent change from baseline in BMD; the Fisher's exact test was used to compare the incidence of new vertebral fractures; and the log‐rank test was used to compare the difference in time to first fracture. The Cox proportional hazards model was used to calculate hazard ratios.

Change in lumbar spine TBS relative to baseline was assessed by generalized estimating equations (adjusted for baseline TBS, treatment, visit, and treatment and visit interaction) and by percentage change from baseline.

Safety evaluations were based on the incidence, severity, and type of AEs and were summarized descriptively. AEs were defined as treatment‐emergent if they occurred on or after the day of administration of the first dose of study drug, if they were considered drug‐related (possibly or probable causality) regardless of when the event occurred, or if they were present at baseline but worsened in severity or were subsequently considered drug‐related by the investigator. In addition, for this particular population, fasting glucose was measured at baseline, day 1, month 1, month 3, month 6, month 9, month 12, and month 18 and change from baseline calculated. HbA_1c_ was not measured.

## Results

### Study population

Of the 2463 patients from the ACTIVE study, 198 were included in the T2DM post hoc analysis (65 in the abaloparatide group, 65 in the placebo group, and 68 in the teriparatide group) and 182 were eligible for inclusion in the TBS analysis. Overall, 77% of patients included in the T2DM post hoc analysis had prior medication for diabetes and 50% had elevated fasting glucose (≥7.0 mmol/L [126 mg/dL]) at baseline (Table 1). The proportion of participants with elevated fasting glucose was similar across groups (48% abaloparatide, 51% placebo, and 50% teriparatide).

**Table 1:** Baseline Characteristics of Patients With T2DM in ACTIVE (ITT Population)

Baseline characteristic	Abaloparatide (*n* = 65)	Placebo (*n* = 65)	Teriparatide (*n* = 68)
Age, years			
Median (range)	70 (58–83)	70 (60–81)	69 (55–84)
Mean (SD)	70.9 (5.2)	70.6 (5.3)	69.2 (6.4)
Time since menopause, years			
Mean (SD)	23.2 (8.5)	21.3 (7.7)	21.0 (7.1)
Weight, kg			
Mean (SD)	60.3 (11.7)	62.4 (11.0)	62.9 (12.5)
BMI, kg/m^2^			
Mean (SD)	25.7 (4.0)	26.4 (3.7)	26.4 (4.2)
Race, *n* (%)			
White	40 (61.5)	45 (69.2)	47 (69.1)
Asian	21 (32.3)	16 (24.6)	17 (25.0)
Black or African American	4 (6.2)	4 (6.2)	3 (4.4)
Other	0	0	1 (1.5)
Prior medication with an indication for diabetes, *n* (%)	51 (78.5)	54 (83.1)	48 (70.6)
Acarbose	2 (3.1)	1 (1.5)	0
DPP‐4 inhibitors	1 (1.5)	1 (1.5)	1 (1.5)
DPP‐4 inhibitors + Metformin	2 (3.1)	1 (1.5)	2 (2.9)
Insulin	2 (3.1)	11 (16.9)	7 (10.3)
Metformin	39 (60.0)	40 (61.5)	35 (51.5)
Repaglinide	0	0	1 (1.5)
Sulfonylurea	28 (43.1)	27 (41.5)	15 (22.1)
Sulfonylurea + Metformin	0	0	1 (1.5)
Thiazolidinediones	0	1 (1.5)	1 (1.5)
Serum creatinine (μmol/L)			
Mean (SD)	60.5 (17.1)	59.5 (14.5)	60.0 (16.0)
Serum creatinine clearance, Cockcroft–Gault formula (mL/min)			
Mean (SD)	77.5 (28.5)	79.4 (23.2)	82.9 (30.2)
Parathyroid hormone (pg/mL)			
Mean (SD)	43.4 (11.6)	41.4 (11.6)	42.8 (9.7)
Fasting glucose ≥126 mg/dL (7.0 mmol/L), *n* (%)	31 (47.7)	33 (50.8)	34 (50.0)
Fasting glucose, mmol/L			
Mean (SD)	7.2 (1.7)	7.5 (2.1)	7.0 (1.8)
Median (min, max)	6.8 (4.0, 13.1)	7.1 (4.4, 17.0)	7.0 (3.1, 14.9)
BMD *T*‐score, mean (SD)			
Femoral neck	−2.2 (0.7)	−2.1 (0.7)	−2.2 (0.8)
Total hip	−1.9 (0.8)	−1.8 (0.8)	−1.9 (0.9)
Lumbar spine	−2.9 (0.9)	−2.9 (0.7)	−2.6 (1.2)
Lumbar spine TBS^a^			
Mean (SD)	1.17 (0.14)	1.21 (0.11)	1.21 (0.09)
Prevalent vertebral fracture, n (%)	11 (16.9)	11 (16.9)	17 (25.0)
≥1 prior nonvertebral fracture within 5 years prior to randomization, *n* (%)	19 (29.2)	17 (26.2)	21 (30.9)
No prior fractures, *n* (%)	31 (47.7)	28 (43.1)	25 (36.8)

ITT = intent‐to‐treat; T2DM = type 2 diabetes mellitus; TBS = trabecular bone score; TPTD = teriparatide.

a For lumbar spine TBS at baseline, *n* = 57 for abaloparatide, *n* = 60 for placebo, and *n* = 65 for TPTD.

Baseline characteristics were generally well‐balanced between treatment groups. Median age was 70 years in the abaloparatide group, 70 years in the placebo group, and 69 years in the teriparatide group. Mean baseline femoral neck BMD *T*‐score was −2.2 in the abaloparatide group, −2.1 in the placebo group, and − 2.2 in the teriparatide group (Table 1). Within 5 years prior to randomization, 29.2% (abaloparatide), 26.2% (placebo), and 30.9% (teriparatide) of patients had at least one prior nonvertebral fracture. Proportionally more patients in the teriparatide group (25.0%) had a prevalent vertebral fracture at baseline compared with those in the abaloparatide (16.9%) and placebo (16.9%) groups. Proportionally fewer patients in the teriparatide group (36.8%) had no prior fractures compared with those in the abaloparatide (47.7%) and placebo (43.1%) groups.

### Change in BMD during ACTIVE in patients with T2DM

**Figure 1:**
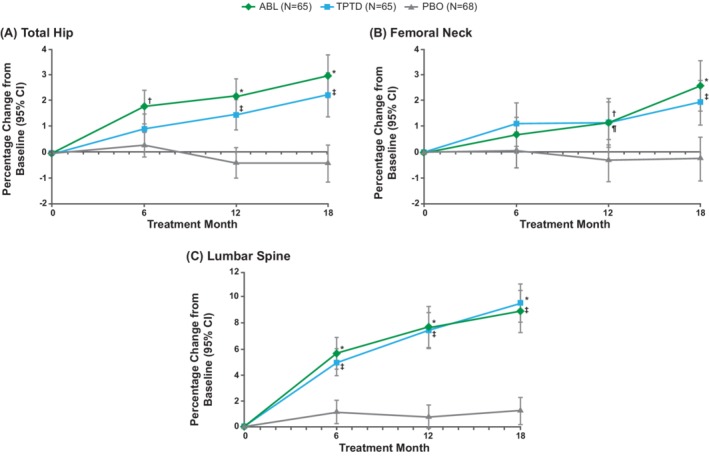
Change in BMD relative to baseline during ACTIVE (**A**baloparatide **C**omparator **T**rial **I**n **V**ertebral **E**ndpoints) for patients with type 2 diabetes mellitus (intent‐to‐treat population) mean percent change in BMD as measured using DXA at the (*A*) total hip, (*B*) femoral neck, and (*C*) lumbar spine in patients from ACTIVE with type 2 diabetes mellitus treated with placebo, abaloparatide, or teriparatide. Improvements in BMD were significantly greater (*p* < 0.05) in the abaloparatide group versus the placebo group at all three sites and at all three time points, except for the femoral neck at 6 months. Improvements in BMD with teriparatide were significantly greater (*p* < 0.05) versus those with placebo at all three sites and all three time points, except for total hip and femoral neck at 6 months. Error bars indicate 95% CIs. Missing BMD data were imputed using the method of last observation carried forward (LOCF). ^*^ *P*<0.001 ABL vs PBO; ^†^ *P*<0.05 ABL vs PBO; ^‡^ *P*<0.001 TPTD vs PBO; ^¶^ *P*<0.05 TPTD vs PBO. ABL, abaloparatide; BMD, bone mineral density; T2DM, type 2 diabetes mellitus; ITT, intent to treat; PBO, placebo; TPTD, teriparatide.

Significantly greater (*p* < 0.05) improvements in total hip, femoral neck, and lumbar spine BMD were observed in the T2DM abaloparatide group versus placebo group at all three time points (6, 12, and 18 months), except for femoral neck at 6 months (Fig. 1). At 18 months, the least squares mean (LSM) difference in change in BMD from baseline between the abaloparatide group and placebo group was 3.3% (95% CI, 2.4 to 4.3) at total hip, 2.8% (95% CI, 1.7 to 4.0) at the femoral neck, and 7.6% (95% CI, 5.9 to 9.3) at the lumbar spine (*p* < 0.001 at all three sites).

Significantly greater (*p* < 0.05) improvements in BMD also were seen at all three sites and at all time points in the teriparatide group versus placebo group, except for total hip and femoral neck at 6 months (Fig. 1). At 18 months, the LSM difference in change in BMD from baseline between the teriparatide group and placebo group was 2.7% (95% CI, 1.7 to 3.6) at total hip, 2.2% (95% CI, 1.0 to 3.4) at the femoral neck, and 8.4% (95% CI, 6.8 to 10.0) at the lumbar spine (*p* < 0.001 at all three sites).

The differences in total hip BMD, femoral neck BMD, and lumbar spine BMD were not significant between the abaloparatide and teriparatide groups at any time point.

### Change in TBS during ACTIVE in patients with T2DM

**Figure 2:**
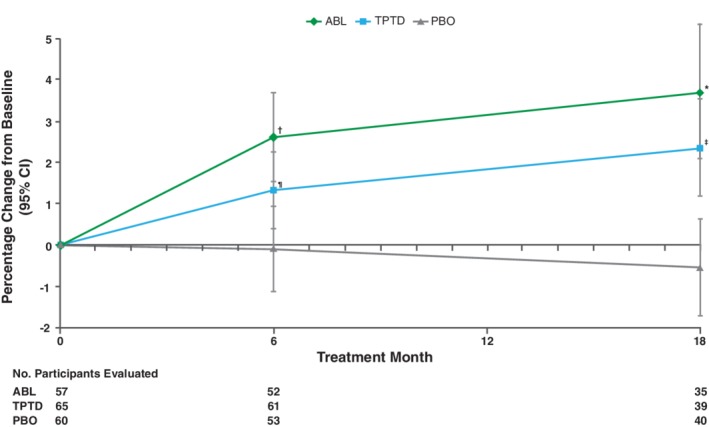
Change in lumbar spine TBS relative to baseline during ACTIVE (**A**baloparatide **C**omparator **T**rial **I**n **V**ertebral **E**ndpoints) in patients with type 2 diabetes mellitus (T2DM) mean percent change from baseline in lumbar spine TBS in patients from ACTIVE with T2DM treated with placebo, abaloparatide, or teriparatide. Significant increases in TBS were seen with abaloparatide versus placebo (*p* < 0.01 at 6 months and *p* < 0.001 at 18 months) and teriparatide versus placebo (*p* < 0.05 at 6 months and *p* < 0.001 at 18 months). Error bars indicate 95% CIs. ^*^ *P*<0.001 ABL vs PBO; ^†^ *P*<0.01 ABL vs PBO; ^‡^ *P*<0.001 TPTD vs PBO; ^¶^ *P*<0.05 TPTD vs PBO. ABL, abaloparatide; PBO, placebo; TBS, trabecular bone score; TPTD, teriparatide.

TBS changes from baseline in patients with T2DM are shown in Fig. 2. At 6 months, mean percent change from baseline in lumbar spine TBS was 2.63% (95% CI, 1.54% to 3.72%) in the abaloparatide group, −1.32% (95% CI, 0.38% to 2.26%) in the teriparatide group, and − 0.10% (95% CI, −1.14% to 0.94%) in the placebo group (*p* < 0.01 for abaloparatide versus placebo and *p* < 0.05 for teriparatide versus placebo; the difference between abaloparatide and teriparatide was not significant). At 18 months, mean percent change from baseline in lumbar spine TBS was 3.72% (95% CI, 2.08% to 5.37%) in the abaloparatide group, 2.37% (95% CI, 1.19% to 3.55%) in the teriparatide group, and − 0.56% (95% CI, −1.74% to 0.62%) in the placebo group. Changes in TBS were significantly different for both abaloparatide and teriparatide versus placebo at 18 months (*p* < 0.001). TBS changes were numerically greater for abaloparatide than for teriparatide at both 6 and 18 months, although the differences were not statistically significant.

### Fracture incidence during ACTIVE in patients with T2DM

No new vertebral fractures occurred in patients with T2DM treated with abaloparatide (0.0%, *n* = 0) or teriparatide (0.0%, *n* = 0), whereas new vertebral fractures occurred in 5.4% (*n* = 3) of patients in the placebo group. Differences were not statistically significant among the T2DM groups.

There were 2 patients with clinical fracture and no patients with a nonvertebral, major osteoporotic, or wrist fracture in the abaloparatide group during the study. In the placebo group, there were 4 patients with nonvertebral fracture, 4 patients with clinical fracture, 3 patients with major osteoporotic fracture, and 1 patient with wrist fracture. In the teriparatide group, there were 2 patients with nonvertebral fracture, 3 patients with clinical fracture, 1 patient with major osteoporotic fracture, and 1 patient with wrist fracture. The Kaplan–Meier estimated event rate for clinical fractures at 19 months was 3.6% in the abaloparatide group, 7.4% in the placebo group, and 5.0% in the teriparatide group. The Kaplan–Meier estimated event rate for nonvertebral fracture, major osteoporotic fracture, and wrist fracture was 7.4%, 5.5%, and 2.0%, respectively, in the placebo group, and 3.2%, 1.5%, and 1.5%, respectively, in the teriparatide group. Differences were not statistically significant except for nonvertebral fractures for abaloparatide versus placebo in patients with T2DM (abaloparatide versus placebo, *p* = 0.04).

No interactions between T2DM status (as determined based on standardized MedDRA preferred terms in patient medical records) and the treatment effect of abaloparatide on fractures or BMD increases were observed.

### Safety and AEs during ACTIVE in patients with T2DM

Mean changes from baseline in clinical laboratory assessments of glucose in patients with T2DM ranged from −0.14 to 0.28 mmol/L in the abaloparatide group, −0.26 to 0.19 mmol/L in the placebo group, and − 0.69 to 0.20 mmol/L in the teriparatide group over the 18‐month study. Overall, mean change in fasting blood glucose (FBG) from baseline at 18 months was 0.03 mmol/L in the abaloparatide group (mean FBG of 7.28 mmol/L at 18 months), 0.09 mmol/L in the placebo group (mean FBG of 7.62 mmol/L at 18 months), and 0.20 mmol/L in the teriparatide group (mean FBG of 7.17 mmol/L at 18 months). Median FBG was within the American Diabetes Association recommended preprandial glucose target range (4.4 to 7.2 mmol/L)^36^ at all time points in all three treatment groups.

The proportion of ACTIVE study patients with T2DM with treatment‐emergent AEs (TEAEs) was 90.8% (*n* = 59) in the abaloparatide group, 93.8% (*n* = 61) in the placebo group, and 97.1% (*n* = 66) in the teriparatide group. The most frequently observed AEs (occurring in ≥5% of patients in any single study group) in patients with T2DM from ACTIVE treated with abaloparatide were similar to those for patients treated with abaloparatide in the overall ACTIVE population^30^ and included hypercalciuria, upper respiratory tract infection, back pain, arthralgia, dizziness, hypertension, and urinary tract infection (Table 2).

**Table 2:** Adverse Events Occurring in ≥5% of Patients With T2DM in ACTIVE

	Abaloparatide	Placebo	Teriparatide
	(*n* = 65)	(*n* = 65)	(*n* = 68)
Most frequently observed adverse events^a^, *n* (%)			
Hypercalciuria	16 (24.6)	9 (13.8)	18 (26.5)
Upper respiratory tract infection	10 (15.4)	5 (7.7)	6 (8.8)
Arthralgia	8 (12.3)	4 (6.2)	9 (13.2)
Back pain	8 (12.3)	9 (13.8)	5 (7.4)
Hypertension	7 (10.8)	3 (4.6)	4 (5.9)
Urinary tract infection	7 (10.8)	2 (3.1)	4 (5.9)
Dizziness	7 (10.8)	5 (7.7)	2 (2.9)
Anemia	6 (9.2)	1 (1.5)	5 (7.4)
Osteoarthritis	5 (7.7)	3 (4.6)	1 (1.5)
Contusion	4 (6.2)	1 (1.5)	2 (2.9)
Muscle spasms	4 (6.2)	2 (3.1)	1 (1.5)
Palpitations	4 (6.2)	0 (0.0)	1 (1.5)
T2DM^b^	4 (6.2)	2 (3.1)	0 (0.0)
Pain in extremity	3 (4.6)	5 (7.7)	7 (10.3)
Constipation	3 (4.6)	6 (9.2)	5 (7.4)
Nephrolithiasis	3 (4.6)	0 (0.0)	4 (5.9)
Cough	3 (4.6)	4 (6.2)	1 (1.5)
Hypertriglyceridemia	2 (3.1)	5 (7.7)	2 (2.9)
Musculoskeletal pain	2 (3.1)	4 (6.2)	1 (1.5)
Nasopharyngitis	2 (3.1)	4 (6.2)	1 (1.5)
Creatinine renal clearance increased	1 (1.5)	0 (0.0)	6 (8.8)
Hypercalcemia	1 (1.5)	0 (0.0)	5 (7.4)
Influenza	1 (1.5)	5 (7.7)	3 (4.4)
Abdominal pain upper	1 (1.5)	4 (6.2)	1 (1.5)
Vertigo	0 (0.0)	0 (0.0)	5 (7.4)
Hypercalcemia (prespecified safety endpoint)^c^, *n/N* (%)	3/65 (4.6)	1/65 (1.5)	8/67 (11.9)

ACTIVE = **A**baloparatide **C**omparator **T**rial **I**n **V**ertebral **E**ndpoints; T2DM = type 2 diabetes mellitus.

a Indicates adverse events that occurred in at least 5% of patients (in any arm) with T2DM in ACTIVE.

b Four patients with a reported adverse event of T2DM were marked “condition aggravated.” The remaining 4 were included in the analysis based on a medical history of “blood glucose increased” or “glucose tolerance impaired” (included under the narrow preferred terms under “hyperglycemia/new onset diabetes mellitus [SMQ]” [MedDRA version 17.1] used to select the T2DM population), not “type 2 diabetes mellitus.”

c Hypercalcemia defined as albumin‐corrected serum calcium value ≥10.7 mg/dL (≥2.67 mmol/L) at any time point, which was a prespecified secondary endpoint.

A total of 11 (16.9%), 9 (13.8%), and 6 (8.8%) patients with T2DM in the abaloparatide, placebo, and teriparatide groups, respectively, had at least one serious TEAE. Similar to what was seen in the ACTIVE study, there were proportionally more AEs leading to study discontinuation in the T2DM subpopulation treated with abaloparatide (6.2%, *n* = 4) compared with those treated with placebo (4.6%, *n* = 3) or teriparatide (4.4%, *n* = 3).

## Discussion

Existing evidence suggests that the increased fracture risk seen in patients with T2DM may occur in the presence of normal or higher than expected BMD and is a consequence of multiple mechanisms, including altered bone metabolism and compromised bone microarchitecture.^16^, ^17^ Specifically, T2DM has been associated with decreased bone turnover, reduced parathyroid hormone levels, and a decrease in circulating markers of bone formation.^15^, ^16^, ^17^, ^25^ Abaloparatide acts through the PTH1R to stimulate bone formation and improve bone microarchitecture, suggesting it may be beneficial for the reduction of fracture risk in patients with T2DM.^27^, ^31^, ^32^, ^33^

This post hoc analysis is the first study to examine the effect of daily s.c. abaloparatide administration in patients with osteoporosis and T2DM. In patients from ACTIVE with T2DM, treatment with abaloparatide significantly increased BMD at all three sites (total hip, femoral neck, and lumbar spine) compared with placebo. This post hoc analysis was underpowered to assess fracture risk reduction; however, numerical reductions in all fracture types were observed. The most frequently observed TEAEs in the T2DM population treated with abaloparatide were consistent with what was seen in the overall ACTIVE population. These findings are consistent with results from the ACTIVE trial and show no evidence that T2DM status impacts the treatment effect or safety profile of abaloparatide.

In light of the evidence that compromised bone microarchitecture contributes to an increased fracture risk in the T2DM population, we also examined the impact of abaloparatide on TBS, which is correlated with and may be a surrogate measure for bone microarchitecture. Our findings showed significant improvement in lumbar spine TBS, suggesting that abaloparatide improves bone microarchitecture in patients with osteoporosis and T2DM. Results for TBS outcomes in the full ACTIVE cohort are not available for comparison with our findings. However, these findings are consistent with previous clinical data showing that abaloparatide significantly improves skeletal microarchitecture, as assessed by TBS, in postmenopausal women with osteoporosis.^33^ TBS is impacted by high BMI, and the adjustment in TBS for BMI is optimized when BMI is between 15 and 35 kg/m^2^.^37^ In this study population, the BMI cut‐off was 33 kg/m^2^, which is well within the acceptable range, and mean baseline BMI was balanced between treatment groups.

Teriparatide was also associated with a significant increase in BMD compared with placebo in patients with T2DM from ACTIVE. These findings are consistent with a previous observational study that reported an increase in BMD in patients with T2DM initiating teriparatide from the **D**irect **A**nalysis of **N**onvertebral Fractures in the **C**ommunity **E**xperience (DANCE) osteoporosis study.^38^ In our study, a numerical reduction in nonvertebral fracture in individuals with T2DM treated with teriparatide compared with placebo was observed, but the difference was not statistically significant. This result is difficult to interpret given the small number of fractures.

Teriparatide treatment also improved lumbar spine TBS, consistent with previous studies in broader populations.^33^, ^39^ In our study, abaloparatide appeared to have a more rapid effect on lumbar spine TBS compared with teriparatide at 6 months, which suggests a potential clinical advantage for abaloparatide. Further, the effect of teriparatide on lumbar spine TBS was numerically less than that with abaloparatide at both 6 and 18 months, but did not reach statistical significance at either time point. In a phase 2 study by Bilezikian and colleagues, a significantly greater increase in TBS was observed in nondiabetic postmenopausal women treated with abaloparatide versus teriparatide.^33^ Additional prospective studies in a larger population of patients with T2DM and osteoporosis are needed to fully elucidate any differences between abaloparatide and teriparatide in terms of effects on TBS.

Limitations of this study include that it was a post hoc subgroup analysis and consideration for sample size for the subset of patients with T2DM was not made a priori. As such, the study had limited statistical power because of the relatively small subset of patients with T2DM from ACTIVE, which restricted the ability to detect changes in fracture incidence in this population. In addition, determination of T2DM status for inclusion in the analysis was based on standardized MedDRA preferred terms. It is possible diabetes status was misclassified. Some participants classified as “no diabetes” may have had undiagnosed diabetes. Also, participants without diabetes or with prediabetes may have been misclassified as having diabetes and included in our analyses. In addition, information on the level of glycemic control in the patients with T2DM included in the analysis was limited to fasting blood glucose as HbA1c was not measured. Further, patients with BMI ˃33 kg/m^2^ were excluded from the ACTIVE study. We acknowledge that this limits the ability to generalize these results to those who are obese in the diabetes populations. The study also only examined osteoporotic postmenopausal women with T2DM and may not be generalizable to those without osteoporosis or other populations. Furthermore, it is not known how long the benefits of abaloparatide observed in this study would extend beyond the 18‐month study period. Finally, teriparatide was administered open‐label. Because study participants and investigators were aware of teriparatide treatment, reporting bias for subjective measures may have occurred.

## Conclusions

In postmenopausal women with both osteoporosis and T2DM, abaloparatide treatment resulted in significant improvements in BMD compared with placebo, consistent with the overall ACTIVE population. Similar results were seen for teriparatide versus placebo. Significant improvement was observed in lumbar spine TBS, which tends to be impaired in the T2DM population, for both abaloparatide and teriparatide, suggesting that these treatments also improved bone microarchitecture.

## Disclosure

RD has participated in a scientific advisory board for Ultragenyx. DH is co‐owner of the trabecular bone score patent and is a part‐time employee of and owns company stock in Medimaps Group. AVS has participated in a scientific advisory board for Amgen and has received a research grant from Hologic. GH is a board member, consultant for and owns stock in Radius Health, Inc. PDM is a consultant to Radius Health, Inc.; has participated on scientific advisory boards for AgNovos, Alexion, Amgen, Eli Lilly, Merck, Radius Health, Inc., Roche, and Sanofi; and has received research grants from Alexion, Amgen, Boehringer Ingelheim, Immunodiagnostics, Eli Lilly, Merck, Merck Serono, National Bone Health Alliance, Novartis, Novo Nordisk, Roche Diagnostics, and Takeda. RGJ has participated in scientific advisory boards for Amgen, NovoNordisk, Janssen, Eli Lilly, and Astra Zeneca; and has received speaker honoraria from Amgen and Novo Nordisk. BM, YW, and LAF are employees of and own stock in Radius Health, Inc.
