# Erratum for Abaloparatide in Postmenopausal Women With Osteoporosis and Type 2 Diabetes: A Post Hoc Analysis of the ACTIVE Study

**DOI:** 10.1002/jbm4.10414

**Published:** 2020-11-03

**Authors:** Ruban Dhaliwal, Didier Hans, Gary Hattersley, Bruce Mitlak, Lorraine A Fitzpatrick, Yamei Wang, Ann V Schwartz, Paul D Miller, Robert G Josse

**Affiliations:** ^1^ Metabolic Bone Disease Center State University of New York Upstate Medical University Syracuse NY USA; ^2^ Center of Bone Disease, Bones & Joints Department Lausanne University Hospital Lausanne Switzerland; ^3^ Clinical Development Radius Health, Inc. Waltham MA USA; ^4^ Biostatistics Radius Health, Inc. Waltham MA USA; ^5^ Department of Epidemiology and Biostatistics UCSF School of Medicine San Francisco CA USA; ^6^ Research Colorado Center for Bone Research Lakewood CO USA; ^7^ Research St. Michael's Hospital, University of Toronto Toronto CO Canada

This article corrects the following: Abaloparatide in Postmenopausal Women With Osteoporosis and Type 2 Diabetes: A Post Hoc Analysis of the ACTIVE Study

Ruban Dhaliwal, Didier Hans, Gary Hattersley, Bruce Mitlak, Lorraine A Fitzpatrick, Yamei Wang, Ann V Schwartz, Paul D Miller, Robert G Josse

Volume 4, Number 4, Journal of Bone and Research Plus page e10346

JBMR Plus. 2020 Feb 27;4(4):e10346. doi: 10.1002/jbm4.10346. eCollection 2020 Apr.

First published online: https://doi.org/10.1002/jbm4.10346

The authors wish to acknowledge that three corrections have been made. In the Results section, regarding change in trabecular bone score (TBS) during ACTIVE in patients with type 2 diabetes mellitus (T2DM), a negative sign was inadvertently inserted before “1.32” that was incorrect in the following phrase:

“At 6 months, mean percent change from baseline in lumbar spine TBS was 2.63% (95% CI, 1.54% to 3.72%) in the abaloparatide group, 1.32% (95% CI, 0.38% to 2.26%) in the teriparatide group, and −0.10% (95% CI, −1.14% to 0.94%) in the placebo group (*P* < 0.01 for abaloparatide versus placebo and *P* < 0.05 for teriparatide versus placebo; difference between abaloparatide and teriparatide was not significant).”^(^ ^1^ ^)^

In Table 2, a portion of footnote b was not relevant; it has therefore been deleted. Table 2 remains unchanged. The corrected footnote is shown here:

ACTIVE = **A**baloparatide **C**omparator **T**rial **I**n **V**ertebral **E**ndpoints; T2DM = type 2 diabetes mellitus.

^a^Indicates adverse events that occurred in at least 5% of patients (in any arm) with T2DM in ACTIVE.

^b^Four patients with a reported adverse event of T2DM were marked “condition aggravated.”

^c^Hypercalcemia was defined as albumin‐corrected serum calcium value ≥10.7 mg/dL (≥2.67 mmol/L) at any time point, which was a prespecified secondary endpoint.

In Fig. 1, the “*N*s” were reported incorrectly for the teriparatide (TPTD) and placebo (PBO) treatment groups. This error has now been corrected in the revised Fig. 1 shown here.

**Fig 1:**
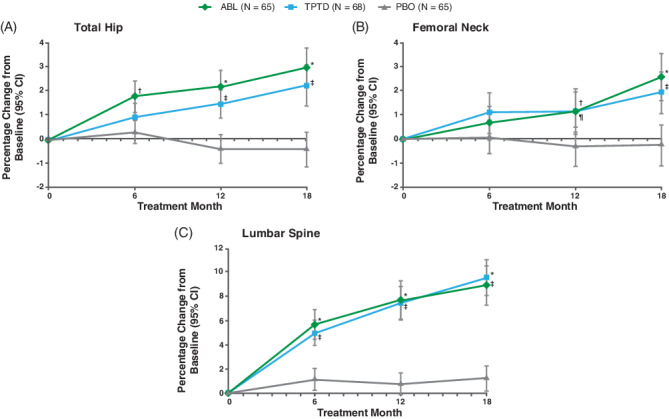
Change in BMD. ABL = abaloparatide; PBO = placebo; TPTD = teriparatide. *P < 0.001 ABL vs PBO; †P < 0.05 ABL vs PBO; ‡P < 0.001 TPTD vs PBO; ¶P < 0.05 TPTD vs PBO.

The authors regret any confusion and inconvenience this may have caused the readers of *JBMR Plus*. The main conclusion of the study, that abaloparatide treatment resulted in significant improvements in BMD and lumbar spine TBS compared with placebo in the T2DM population, was not affected by the errors.

### PEER REVIEW

The peer review history for this article is available at https://publons.com/publon/10.1002/jbm4.10414.
